# Rationally designed naphthyl substituted amine functionalized ionic liquid platform for covalent immobilization and direct electrochemistry of hemoglobin

**DOI:** 10.1038/s41598-019-46982-3

**Published:** 2019-07-18

**Authors:** K. Theyagarajan, Duraisamy Saravanakumar, Sellappan Senthilkumar, Kathavarayan Thenmozhi

**Affiliations:** Department of Chemistry, School of Advanced SciencesVellore Institute of Technology (VIT), Vellore, 632014 India

**Keywords:** Sensors, Electrochemistry

## Abstract

Herein, we have designed and demonstrated a facile and effective platform for the covalent anchoring of a tetrameric hemoprotein, hemoglobin (Hb). The platform comprises of naphthyl substituted amine functionalized gel type hydrophobic ionic liquid (NpNH_2_-IL) through which the heme protein was covalently attached over a glassy carbon electrode (Hb-NpNH_2_-IL/GCE). UV-vis and FT-IR spectral results confirmed that the Hb on NpNH_2_-IL retains its native structure, even after being covalently immobilized on NpNH_2_-IL platform. The direct electron transfer of redox protein could be realized at Hb-NpNH_2_-IL/GCE modified electrode and a well resolved redox peak with a formal potential of −0.30 V and peak separation of 65 mV was observed. This is due to the covalent attachment of highly conducting NpNH_2_-IL to the Hb, which facilitates rapid shuttling of electrons between the redox site of protein and the electrode. Further, the fabricated biosensor favoured the electrochemical reduction of bromate in neutral pH with linearity ranging from 12 to 228 µM and 0.228 to 4.42 mM with a detection limit and sensitivities of 3 µM, 430.7 µA mM^−1^ cm^−2^ and 148.4 µA mM^−1^ cm^−2^ respectively. Notably, the fabricated biosensor showed good operational stability under static and dynamic conditions with high selectivity and reproducibility.

## Introduction

Ionic liquids (ILs) are superior class of non-molecular ionic materials that possesses multifaceted customizable properties. They are fascinating melts of salts, composed of asymmetric or bulky organic cation with organic or inorganic anions that can undergo varieties of structural and functional variations^1–3^. Due to their unique archetypal properties like high ionic conductivity, low volatility, non-flammability, high thermal and chemical stability, tremendous efforts have been taken to synthesis and utilize ILs in diverse fields ranging from academic to industrial research and applications^4–6^. Further, ILs possess very high electrochemical stability over wide potential window, which makes them interesting candidates in variety of electrochemical applications such as supercapacitors^7^, batteries^8^, corrosion inhibitors^9^, electrochemical sensors^10,11^ and biosensors^12,13^. Noticeably, physicochemical properties of ILs can be tuned by judiciously altering the cation and anion or by introducing new functionalities around the cation or anion in order to meet the requirements for the task specific applications^14–16^.

ILs exhibit remarkable biocompatibility because of their ability to form hydrogen bonding and electrostatic interaction with the biomolecules and consequently preventing the unfolding of their secondary structure^17,18^. Thus, ILs are considered as highly pertinent platform for the immobilization and stabilization of enzymes, proteins and living cells, which retains the activity of these biomolecules for a prolonged period^12,19^. However, immobilization by physical adsorption or by entrapment or encapsulation of biomolecules over ILs or other support matrices suffers a drawback of leaching out of biomolecules from the electrode surface and lacks electrical communication during electrochemical applications^20^. In order to overcome these disadvantages, strategies for covalent and irreversible immobilization of biomolecules are continuously being explored^21,22^. A stable chemical bond between the biomolecule and the support matrix creates a firm linkage, thereby preventing it from leaching out of the electrode surface and also improves its electron transfer kinetics. Thus, covalent immobilization of biomolecules on ILs containing suitable reactive functional groups would enhance the electrical contact as well as improve the operational stability^23,24^.

Substrates with amine and carboxylic acid functional groups are widely used for the covalent immobilization of biomolecules^25–27^. Since most of the biomolecules have peptide and amino acid groups, the substrate with amine or carboxylic acid group can be covalently linked via glutaraldehyde cross coupling or through EDC/NHS coupling to form a stable imine or amide bond respectively^25,28^. Hence, design and synthesis of novel ILs with suitable functional group becomes imperative for specific applications, whereas reports on functionalized ILs are very scarce. This motivated us to design various functionalized IL based platforms for covalent immobilization of biomolecules^23^. Herein, our focus is to synthesize an imidazolium based amine functionalized IL platform for the simple and stable anchoring of biomolecules. Naphthyl (Np) group was introduced around the imidazolium unit in order to improve the stability and electrical contact through pi-pi stacking between the naphthyl ring and carbon electrode. Imidazolium based amine functionalized IL prepared with bromide anion was obtained as water soluble solid, and this compound upon anion exchange with triflate (TFSI) anion resulted in the formation of a water insoluble viscous gel, which sticks firmly on the electrode surface. This, naphthyl substituted amine functionalized IL containing TFSI anion (NpNH_2_-IL) was tested as the new platform for the covalent immobilization of biomolecules.

Owing to easy availability, low cost and clear structure, hemoglobin (Hb) has been widely studied as the ideal prototype for investigating direct electron transfer (DET) of redox proteins^29–31^. Since the active sites of Hb are deeply buried inside the thick peptide layers, DET is realized only in high conducting platforms^32,33^. Thus, covalent immobilization of Hb on functionalized IL platform would facilitate the shuttling of electrons from the electroactive centre of the Hb protein to the electrode, and also ensures stable anchoring of Hb in the biosensor setup. In the present work, we have demonstrated the covalent immobilization of Hb on our newly synthesized amine functionalized gel type hydrophobic ionic liquid through terephthaloyl chloride cross-coupling reaction. High ionic conductivity of IL enhances the electrical wiring from protein to electrode, thereby DET of Hb was established. The modified electrode thus fabricated was tested for the biosensing of bromate (BrO_3_^−^), which is a disinfection byproduct during the ozonisation of water and is regarded as the potent carcinogen to humans and animals^34–36^. The biosensor displayed remarkable performance towards the detection of bromate, which is attributed to the simple and effective architect offered by the NpNH_2_-IL platform.

## Methods

### Synthesis of naphthyl substituted amine functionalized IL (NpNH_2_-IL)

**Figure 1:**
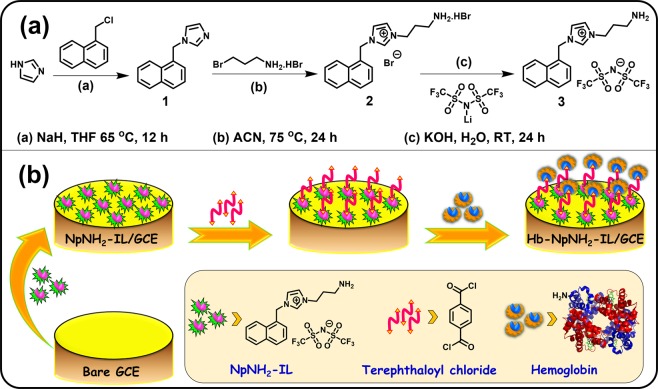
Schematic representations of (**a**) Synthesis of amine functionalized ionic liquid (NpNH_2_-IL). (**b**) Stepwise covalent immobilization of Hb on NpNH_2_-IL platform and the fabrication of Hb-NpNH_2_-IL/GCE modified electrode.

The naphthyl substituted amine functionalized IL to be used as the platform for Hb immobilization has been synthesized through the following steps based on the previous reports with slight modification^37,38^ and a schematic representation is shown in Fig. 1a.

### Synthesis of 1-naphthalen-1-ylmethyl-1H-imidazole (1)

Dry THF suspension of oil-free sodium hydride (21 mmol) was stirred under ice cold condition, to which imidazole (20 mmol) was added dropwise under constant stirring. After completion of addition, stirring was continued for another 2 h without cooling. Subsequently, to the reaction mixture, 1-(chloromethyl)naphthalene (20 mmol) in dry THF was added and refluxed at 65 °C for 12 h. Further, the reaction mixture was filtered, followed by the removal of solvent using rotary evaporation. The residue obtained was washed with water and then extracted thrice with chloroform and the combined extracts were dried over anhydrous sodium sulphate followed by solvent evaporation to give 1-(naphthalen-1-ylmethyl)-1H-imidazole as brown viscous liquid. Yield: 9.58 g (92%). ^1^H NMR (400 MHz, CDCl_3_): *δ* 5.54 (s, 2 H, -CH_2_-), 6.90 (s, 1 H, -CH-), 7.08 (s, 1 H, -CH-), 7.13–7.15 (d, 1 H, -CH-), 7.40–7.43 (t, 1 H, -CH-), 7.50–7.54 (m, 3 H, -CH-), 7.83–7.90 (m, 3 H, -CH-) ppm. ^13^C NMR (100 MHz, CDCl_3_): *δ* 48.67, 119.47, 122.37, 125.46, 126.16, 126.25, 126.98, 129.03, 129.26, 129.68, 130.84, 131.40, 133.78, 137.54 ppm.

### Synthesis of 3-ammoniopropyl-1-(naphthalen-1-ylmethyl)-1H-imidazol-3-ium bromide (2)

An acetonitrile solution of 3-bromopropylamine hydrobromide (15.2 mmol) was slowly added to 1-naphthalen-2-ylmethyl-1H-imidazole (15 mmol) in acetonitrile under constant stirring. Further, this reaction mixture was refluxed at 75 °C under N_2_ atmosphere for 24 h. Next, the solvent was removed by vacuum and the solid residue was washed twice with ethyl acetate and thrice with diethyl ether. Finally, after drying under vacuum for 2 h, 3-ammoniopropyl-1-(naphthalen-1-ylmethyl)-1H-imidazol-3-ium bromide was obtained as white solid. Yield: 3.80 g (89%). ^1^H NMR (400 MHz, DMSO-*d*_6_): *δ* 2.07–2.14 (m, 2 H, -CH_2_-), 2.80 (s, 2 H, -CH_2_-), 4.29–4.32 (t, 2 H, -CH_2_-), 5.97 (s, 2 H, -CH_2_-), 7.56–7.63 (m, 5 H), 7.86–7.87 (dd, 2 H), 7.96 (br s, 3 H, -NH_3_), 8.01–8.03 (d, 2 H), 8.14–8.16 (d, 1 H), 9.40 (s, 1 H, imidazole C2, -CH-) ppm. ^13^C NMR (100 MHz, DMSO-*d*_6_): *δ* 27.99, 36.18, 46.65, 50.40, 123.13, 123.43, 123.47, 126.15, 126.91, 127.71, 128.24, 129.36, 130.13, 130.50, 130.90, 133.89, 137.04 ppm.

### Synthesis of 3-aminopropyl-1-(naphthalen-1-ylmethyl)-1H-imidazol-3-ium bis((trifluoro-methyl)sulfonyl)amide (NpNH_2_-IL) (3)

3-Ammoniopropyl-1-(naphthalen-1-ylmethyl)-1H-imidazol-3-ium bromide was dissolved in minimum of water and the pH was adjusted to 8 by adding small portions of aqueous KOH. Subsequently, equivalent amount of lithium bis(trifluoromethane) sulfonimide in water was introduced slowly and the stirring was continued for 24 h at room temperature. During the course of reaction, two layers were formed: an ionic liquid layer, and an aqueous layer containing lithium bromide salt along with the unreacted starting material. After decanting the top aqueous layer, NpNH_2_-IL with TFSI anion was obtained as brown viscous liquid and the IL was vacuum dried at 90 °C for 12 h. Yield: 1.16 g (85%). ^1^H NMR (400 MHz, DMSO-*d*_6_): *δ* 1.94 (s, 2 H, -CH_2_-), 2.64 (s, 2 H, -CH_2_-), 3.17 (s, 2 H, -NH_2_), 4.23 (s, 2 H, -CH_2_-), 5.94 (s, 2 H, -CH_2_-), 7.53–7.62 (m, 4 H, -CH-), 7.82 (s, 2 H, -CH-), 8.03–8.12 (d, 3 H, -CH-), 9.25 (s, 1 H, imidazole C2, -CH-) ppm. ^13^C NMR (100 MHz, DMSO-*d*_6_): *δ* 30.47, 37.27, 46.98, 50.43, 118.36, 121.56, 123.19, 123.28, 123.46, 126.12, 126.92, 127.67, 128.15, 129.40, 130.17, 130.45, 130.88, 133.93, 136.95 ppm. ^19^F NMR (376 MHz, DMSO-*d*_6_): *δ* −78.72 ppm. HRMS (EI): [C_17_H_20_N_3_]^+^, calculated mass 266.3675, found 266.3672.

### Fabrication of Hb-NpNH_2_-IL/GCE modified electrode

The GCE electrode (3 mm dia.) was mirror polished prior to the modification using alumina slurries on a polishing pad. Further, the GCE was ultrasonically cleaned by sonicating in ethanol:water mixture (1:1) and finally dried under a stream of nitrogen. Typically, 5 µL of hydrophobic gel type NpNH_2_-IL (2 mg in 100 µL of MeOH) was dropcasted on the GCE surface and dried in air. Then, 5 µL of terephthaloyl chloride (2 mg in 100 µL of EtOH) crosslinker was dropped and allowed to react with the -NH_2_ group of the NpNH_2_-IL for 2 h. Finally, 5 µL of Hb (1 mg in 100 µL of phosphate buffer, 0.1 M, pH 7.0) was dropped and incubated for 2 h to complete the crosslinking reaction of -NH_2_ groups of Hb with the crosslinker. The modified electrode was finally rinsed with phosphate buffer to remove the loosely bound and unreacted Hb molecules on the electrode surface to furnish Hb-NpNH_2_-IL/GCE modified electrode. The stepwise fabrication of the modified electrode is shown in Fig. 1b.

## Results and Discussion

The synthesis of naphthyl substituted amine functionalized IL was monitored using ^1^H, ^13^C & ^19^F NMR and HRMS spectral analysis (Supplementary Figures S1–S8) and the spectral data were in accordance with the synthesized compounds. Hb protein was covalently attached on NpNH_2_-IL platform using terephthaloyl chloride crosslinker through amide bond formation.

### Spectroscopic investigations of Hb and Hb-NpNH_2_-IL modified electrodes

**Figure 2:**
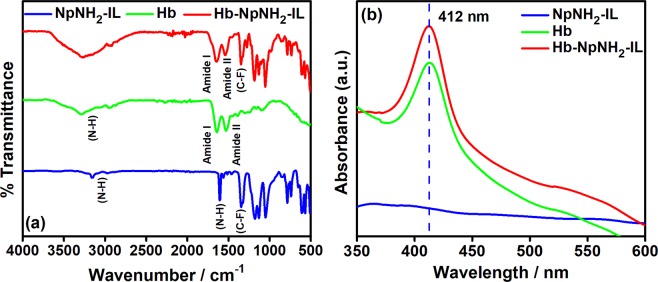
(**a**) FT-IR and (**b**) UV-vis comparison spectra of NpNH_2_-IL (blue), Hb (green) and Hb-NpNH_2_-IL (red) as dried film on a glass substrate obtained at different stages during fabrication.

Covalent immobilization of hemoglobin on amine functionalized ionic liquid was examined using FT-IR and UV-vis spectroscopy. Figure 2a shows the FT-IR spectra of NpNH_2_-IL (blue), native Hb (green) and Hb-NpNH_2_-IL (red). The FTIR spectrum of NpNH_2_-IL (curve a) shows peaks at 3176 and 1604 cm^−1^ corresponding to N-H stretching and bending vibrations of free amino group. The vibrations at 1346 and 1130 cm^−1^ are assigned to C-F and O = S = O of triflate anion, respectively^39,40^. In curve b, C = O stretching vibration of amide I appears at 1643 cm^−1^ and the vibration at 1531 cm^−1^ is ascribed to the vibrations from both N-H and C-N groups of amide II in native protein^41^. In the case of Hb-NpNH_2_-IL (curve c), the peak at 1604 cm^−1^ was found to disappear and appearance of a tiny shoulder with broadening of amide I peak confirms the formation of amide bond between the NpNH_2_-IL and Hb protein via terephthaloyl chloride cross coupling. Moreover, amide I and amide II peaks of native Hb were retained in Hb-NpNH_2_-IL(curve c) with negligible shift in their frequencies, which confirms that the proteins were not denatured after covalent immobilization on NpNH_2_-IL platform.

UV-vis studies were used to monitor any observable changes in the Soret band of the heme moiety. Any deviation in the Soret absorption band would infer possible denaturation of the heme protein or any conformational changes that would have occurred on Hb during the modification. Figure 2b depicts the UV-vis spectra of NpNH_2_-IL (blue), native Hb (green) and Hb-NpNH_2_-IL (red) on quartz glass substrate as dried film. As observed from curve a, NpNH_2_-IL did not show any absorption peak, whereas Hb-NpNH_2_-IL exhibited a Soret absorption band at 412 nm (curve c), similar to that observed for native Hb (curve b)^42^. These results indicate that the secondary structure of Hb is not destroyed even after covalently immobilizing over the NpNH_2_-IL. The UV-Vis and FT-IR results clearly portray that NpNH_2_-IL can be used as a facile matrix for the effective covalent attachment of proteins and other biomolecules.

### Electrochemical impedance spectroscopy (EIS)

**Figure 3:**
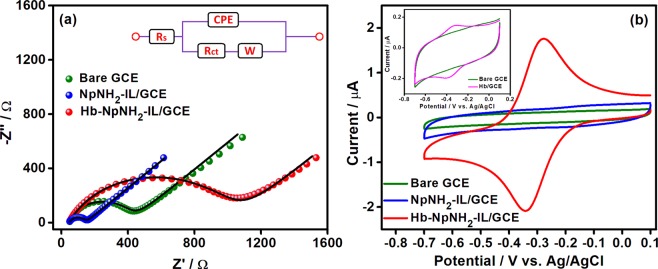
(**a**) Nyquist plot of bare GCE (green), NpNH_2_-IL/GCE (blue), Hb-NpNH_2_-IL/GCE (red) in 5 mM [Fe(CN)_6_]^3−^ solution prepared using 0.1 M KCl. Inset: Equivalent circuit. (**b**) Cyclic voltammograms of bare GCE (green), NpNH_2_-IL/GCE (blue) and Hb-NpNH_2_-IL/GCE (red) in N_2_ saturated phosphate buffer (0.1 M, pH 7.0) at 50 mVs^−1^. Inset: CVs of bare GCE (green) and Hb/GCE (pink) under similar conditions.

Changes that occurred on the surface of the modified electrode at each step during the fabrication of biosensor was probed in through electrochemical impedance spectroscopy. Figure 3a shows the Nyquist plots (*Z*″ imaginary part vs *Z*′ real part) of bare GCE (green), NpNH_2_-IL/GCE (blue) and Hb-NpNH_2_-IL/GCE (red) modified electrodes in the frequency range of 100 kHz −1 Hz in 5 mM [Fe(CN)_6_]^3−^ solution prepared using 0.1 M KCl. The obtained impedance data were fitted to Randles circuit by using the following elements: charge transfer resistance (*R*_ct_), electrolyte solution resistance (*R*_s_), Warburg element (*W*) and constant phase element (*CPE*). The diameter of semicircle in the Nyquist plot represents the *R*_ct_, indicating the electron transfer kinetics of the redox probe, whereas the linear portion corresponds to the diffusion process. Bare GCE showed a large *R*_ct_ of 401 Ω revealing a slow electron transfer on bare GCE. Subsequent deposition of NpNH_2_-IL on GCE, significantly decreases the *R*_ct_ to 104 Ω, which implies that the introduction of highly conductive NpNH_2_-IL on GCE reduces the electron transfer barrier and facilitates good electron shuttling behaviour to NpNH_2_-IL/GCE modified electrode. The *R*_ct_ of Hb-NpNH_2_-IL/GCE increased to a highest value of 1028 Ω, due to the high electron transfer resistance of non-conducting dense polypeptide layers of Hb, which confirms the covalent immobilization of Hb. From the EIS results, it is evident that the immobilization of NpNH_2_-IL on GCE offers a highly conducting environment (low *R*_ct_) through which the DET of enzymes and proteins could be envisioned.

### Direct electrochemistry of Hb on Hb-NpNH_2_-IL/GCE modified electrode

Figure 3b shows the CVs of electrodes obtained during various stages of modification in N_2_ saturated, phosphate buffer (0.1 M, pH 7.0) at a scan rate of 50 mV s^−1^ in the potential range from 0.1 to −0.7 V. At bare GCE and NpNH_2_-IL/GCE modified electrode, no redox peaks were found since both are redox inactive within the given potential range. However, in Hb-NpNH_2_-IL/GCE a well-defined redox peak with a formal potential (*E*°) of −0.30 V and a peak separation (∆*E*_p_) of 65 mV was observed. This redox peak corresponds to Fe^III^/Fe^II^ redox couple of Hb (Equation (1)), which is deeply buried inside the dense polypeptide layers^43,44^. Further, for comparison, Hb/GCE was fabricated through dropcasting of Hb directly on bare GCE. The Hb/GCE was subjected to continuous cycles in similar conditions and the CV obtained for the first cycle is shown as inset to Fig. 3b. A feeble and unstable redox peak at a higher *E*° of −0.36 V was observed for the first cycle and the voltammetric response started diminishing after the first cycle which could be due to the leaching of Hb protein on continuous cycling and denaturation of the protein on bare GCE^44^. This indicates that the DET of Hb is very challenging at bare GCE, whereas it could be achieved successfully at Hb-NpNH_2_-IL/GCE. The good biocompatible and conductive matrix of NpNH_2_-IL provided a suitable microenvironment for the persuasive immobilization of Hb on the electrode through which the DET of Hb was realized.

1 $${\rm{Hb}}\,{\rm{heme}}\,({{\rm{Fe}}}^{{\rm{III}}})+{{\rm{H}}}^{+}+{{\rm{e}}}^{-}\leftrightarrow {\rm{Hb}}\,{\rm{heme}}\,({{\rm{Fe}}}^{{\rm{II}}})\,({Direct}\,{electron}\,{transfer})$$

The CVs of Hb-NpNH_2_-IL/GCE were recorded at varying scan rates from 10 to 400 mV s^−1^ in order to understand the electron transfer characteristics of Hb and are shown in Supplementary Figure S10. Both the anodic (*I*_pa_) and cathodic (*I*_pc_) peak currents increase linearly with the square root of scan rate, which indicates a characteristic diffusion controlled electrochemical behaviour. Also, it could be seen from the plot that the ratio of their slopes (S_ipa_/S_ipc_) was close to 1.

The pH dependence of the fabricated biosensor response in 0.1 M phosphate buffer was studied in the pH ranging from 4.0 to 9.0 (Supplementary Figure S11). Maximum response current was attained in neutral pH (7.0), which is close to the physiological environment and could retain the activity of the biomolecules effectively. Hence, neutral pH was chosen for investigating the direct electrochemistry of Hb. Further, the stability of the fabricated biosensor at various temperatures (15 to 50 °C) was investigated (Supplementary Figure S12). The cathodic peak current increased with the increase in temperature from 15 to 25 °C, after which the response remained stable up to 35 °C. However, when the temperature was increased beyond 35 °C, the current response decreased drastically, which could be due to the denaturing of Hb protein at elevated temperatures. Since, the current response was maximum and similar in the temperature range from 25 to 35 °C, we have chosen 25 °C (room temperature) as the optimum temperature for further experiments.

### Electrocatalytic reduction of bromate at Hb-NpNH_2_-IL/GCE

**Figure 4:**
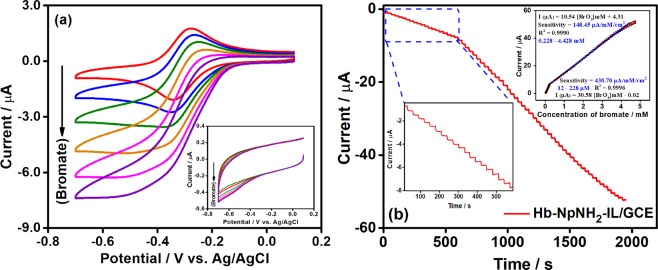
(**a**) CVs obtained for Hb-NpNH_2_-IL/GCE with increasing (0.2, 0.4, 0.6, 0.8, 1.0 mM) concentrations of bromate at 50 mVs^−1^. Inset: NpNH_2_-IL/GCE for the same concentrations of bromate (**b**) Amperometric response of Hb-NpNH_2_-IL/GCE at −0.4 V for the sequential additions of varying concentrations of bromate into a continuously stirred (350 rpm) 0.1 M phosphate buffer (0.1 M, pH 7.0, N_2_ saturated). Inset: Corresponding calibration plot.

It is well known that enzymes or proteins which have bioactive heme protein possess high electrocatalytic activity. After successfully realizing the DET of Hb at the newly synthesized NpNH_2_-IL based platform, our next attempt was to explore the electrocatalytic activity of Hb-NpNH_2_-IL/GCE modified electrode. Bromate is a stable anion with high solubility in water and is difficult to be degraded naturally^45^. Hence, it is highly essential to detect and estimate the amount of bromate present in drinking water and other water bodies. Accordingly, we intended to utilize the Hb-NpNH_2_-IL/GCE towards the electrocatalytic reduction and selective determination of bromate. The CVs were recorded in a N_2_ saturated phosphate buffer (0.1 M, pH 7.0) at 50 mV s^−1^ and are shown in Fig. 4a. As shown in the inset, the NpNH_2_-IL/GCE (in the absence of Hb) showed poor current response for the reduction of bromate. Noticeably, the cathodic peak current of Hb-NpNH_2_-IL/GCE increased linearly for every addition of bromate concentration, indicative of the typical electrocatalytic behaviour at Hb in the NpNH_2_-IL platform. On applying the reduction potential to Hb-NpNH_2_-IL/GCE, Hb (Fe^III^) gets reduced to Hb (Fe^II^) at the modified electrode and the reduced form of Hb (Fe^II^) further reduces the bromate in the solution to bromide and gets re-oxidized to Hb (Fe^III^). The electrocatalytic behaviour of Hb-NpNH_2_-IL/GCE towards bromate is represented by the following Equation (2),

2 $${\rm{Hb}}\,{\rm{heme}}\,({{\rm{Fe}}}^{{\rm{II}}})+{{{\rm{BrO}}}_{{\rm{3}}}}^{-}+{{\rm{6H}}}^{+}\to {\rm{Hb}}\,{\rm{heme}}\,({{\rm{Fe}}}^{{\rm{III}}})+{{\rm{Br}}}^{-}+{{\rm{3H}}}_{{\rm{2}}}{\rm{O}}$$

Amperometric measurements were performed in order to examine the efficacy of the Hb biosensor under dynamic conditions as well as to evaluate the widest possible linear working range for the fabricated biosensor. The convective transport of analyte was obtained by stirring the solution at 350 rpm constantly using a magnetic stirrer. Figure 4b shows the amperometric response obtained for Hb-NpNH_2_-IL/GCE modified electrode at −0.4 V in N_2_ saturated phosphate buffer (0.1 M, pH 7.0). For every addition of bromate, there was a rapid increase in the catalytic current which gets stabilized in less than 3 s. Linear response was achieved in the range from 12 µM to 228 µM with a sensitivity of 430.7 µA mM^−1^ cm^−2^ and 0.228 mM to 4.42 mM with a sensitivity of 148.4 µA mM^−1^ cm^−2^ and detection limit (S/N = 3) was calculated as 3 µM. Analytical parameters obtained were better than or comparable with the recently reported literature on electrochemical bromate detection (Table 1). Wide linear range with rapid response and high sensitivity of the fabricated biosensor could be due to the high conductivity along with the remarkable biocompatibility offered by the NpNH_2_-IL for the effective covalent immobilization of Hb.

**Table 1:** Analytical performances of various electrochemical bromate sensors.

Electrode	Linear range	Sensitivity (µA µM^−1^ cm^−2^)	LOD (µM)	Ref
ERGO–PANOA–Pd/GCE	4–840 µM	0.46	1.0	^46^
GRA/GO/PEI-PANI-PMo_12_	7.5–500 µM	—	3.0	^47^
NENU-3	50–19100 µM and 19.1–72.74 mM	11.2 and 5.08	12.0	^48^
PMo_12_@rGO-PDDA/GCE	20–10000 µM	454	—	^34^
Ag/MWNTs/GCE	500–4000 µM and 4.0–20.0 mM	580.33 and 121.44	—	^49^
PMo_12_/PEDOT/AuNP/GC	250–3000 µM	68	—	^50^
Hb/f-MWCNT-P-L-His-ZnO/GCE	2–15000 µM	35.71	0.3	^51^
MWCNT-PLL/Hb/GCE	15–6000 µM	108	0.96	^52^
Graphene/B-CD/Hb/GCE	0.1–176.6 µM	3.39	0.03	^53^
Hb-NpNH_2_-IL/GCE	12–228 µM and 0.228–4.42 mM	430.70 and 148.45	3.0	This work

### Stability, reproducibility and selectivity of Hb-NpNH_2_-IL/GCE biosensor

**Figure 5:**
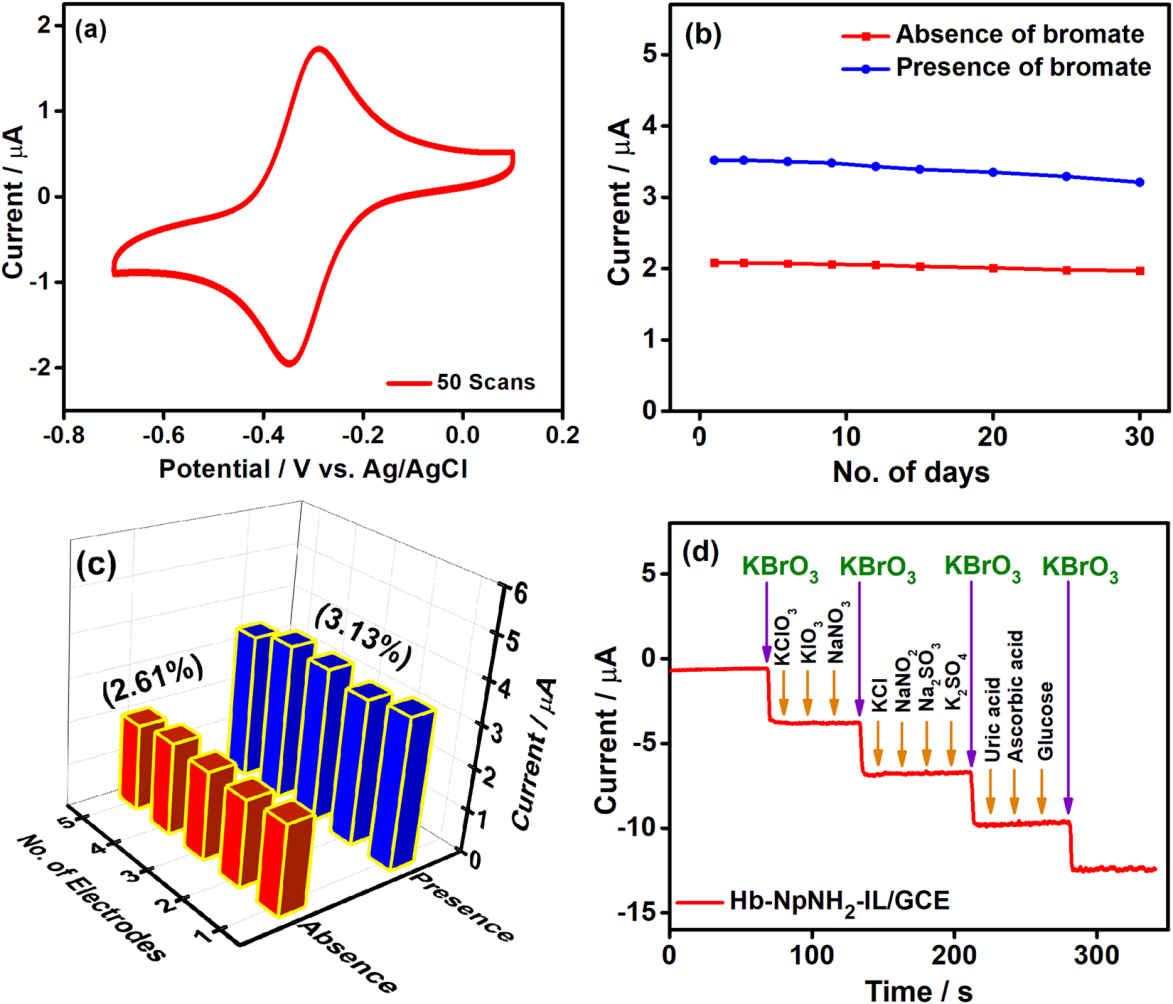
(**a**) CVs of 50 continuous potential cycles of Hb-NpNH_2_-IL/GCE at 50 mV s^−1^. (**b**) Day-to-day current obtained for Hb-NpNH_2_-IL/GC over 30 days (**c**) Columnar diagram of current response obtained for five individually prepared Hb-NpNH_2_-IL/GCE without (red) and with (blue) 0.5 mM bromate. (**d**) Amperometric response (−0.4 V) of Hb-NpNH_2_-IL/GCE for the successive additions of 0.1 mM bromate and 0.5 mM of commonly interfering species in a stirred solution. Electrolyte: N_2_ saturated phosphate buffer (0.1 M, pH 7.0).

The operational stability of the designed biosensor was examined and its amperometric response in the presence of 1.5 mM bromate at −0.4 V in a constantly stirred (350 rpm) phosphate buffer (0.1 M, pH 7.0) are shown in Supplementary Figure S13. It could be seen that the fabricated biosensor showed a stable response for more than 1300 s under same condition in the presence of bromate. Further, the Hb-NpNH_2_-IL/GCE was subjected to continuous potential cycles and there is no obvious change in the peak potentials as well as peak currents even after 50 cycles in the potential range studied (Fig. 5a). Moreover, the long-term stability of Hb biosensor was evaluated for a period of 30 days at regular intervals in the presence as well as absence of 0.5 mM bromate concentration. In Fig. 5b, the modified electrode did not show any significant change in its performance and 95% of its current response was retained in the absence of bromate and 91% in the presence of bromate even after 30 days. The promising operational steadiness and good long-term stability of Hb biosensor could be ascribed to the effective covalent binding of Hb to NpNH_2_-IL. Also, the IL platform holds the protein effectively on the electrode surface and retains its nativity to offer enhanced biocompatibility for a prolonged period. The reproducibility of the biosensor was investigated by freshly constructing five different Hb-NpNH_2_-IL/GC electrodes and evaluated their current responses under identical conditions. The cathodic peak current was recorded in the absence and presence of 0.5 mM bromate (Fig. 5c) and the biosensor showed a relative standard deviation of 2.61% and 3.13% respectively, revealing the excellent reproducibility among the freshly fabricated biosensors.

Further, the selectivity of the biosensor towards the target analyte was tested in the presence of commonly interfering species such as KClO_3_, KIO_3_, NaNO_3_, KCl, NaNO_2_, Na_2_SO_3_, K_2_SO_4_, uric acid, ascorbic acid and glucose in 0.1 M phosphate buffer. Figure 5d shows the amperometric response obtained for the biosensor during successive addition of 0.1 mM bromate and the biosensor showed a rapid response for every addition of bromate. However, there is no significant response seen for the addition of 5-fold excess concentrations of the interfering species under the same condition. Thus, the fabricated biosensor demonstrated good selectivity for bromate reduction over other species, and hence Hb-NpNH_2_-IL/GCE can be employed for the reduction of bromate in real samples.

### Applicability of biosensor in real sample analysis

The practical applicability of the biosensor is the crucial parameter to evaluate the effectiveness of the biosensor in determining the analyte in real samples. Accordingly, the fabricated Hb-NpNH_2_-IL/GCE was tested for the determination of bromate in different water samples (Supplementary Figure S14). Bromate of known concentrations were spiked by following standard addition method and their recoveries were monitored at −0.4 V in amperometry under constant stirring. Obtained recoveries ranged from 98% to 102% and the values are listed in Table 2. These results validate the practicability of the fabricated biosensor for the effective determination of bromate present in real samples.

**Table 2:** Analysis of bromate spiked in real samples.

Sample	Spiked (mM)	Found (mM)	Recovery (%)	RSD (%)
Tap water	0.05	0.051	102.0	2.8
	0.15	0.151	100.6	3.1
Drinking water	0.10	0.102	102.0	2.2
	0.20	0.197	98.5	2.4

## Conclusion

In this report, a highly facile biosensor was constructed using the rational architect of a naphthyl substituted amine functionalized IL over GCE, to achieve the direct electrochemistry of Hb. The hydrophobic gel type NpNH_2_-IL offered high ionic conductivity and impressive biocompatibility for the effective immobilization of Hb. Hb was covalently immobilized to the amino group of NpNH_2_-IL, which provided a rigid, leak-free and highly stable platform. The fabricated biosensor was very promising towards the detection of bromate with high sensitivity and selectivity and a wide linear working range. By virtue of its simplicity, synthetic versatility, biocompatibility, ease of fabrication and high conductivity, the NpNH_2_-IL demonstrates itself as an efficient scaffold for the covalent immobilization of living cells and other biomolecules, which could open diverse channels in bioanalytical device fabrication.

## Supplementary information

Rationally designed naphthyl substituted amine functionalized ionic liquid platform for covalent immobilization and direct electrochemistry of hemoglobin

## Data Availability

The data that support the findings of this research work are available from the corresponding author upon request.

## References

[1] Hapiot P, Lagrost C (2008). Electrochemical reactivity in room-temperature ionic liquids. Chem. Rev..

[2] Vadahanambi S, Jung J, Kumar R, Kim H, Oh I (2013). An ionic liquid-assisted method for splitting carbon nanotubes to produce graphene nano-ribbons by microwave radiation. Carbon.

[3] Damlin P, Suominen M, Heinonen M, Kvarnstrom C (2015). Non-covalent modification of graphene sheets in PEDOT composite materials by ionic liquids. Carbon.

[4] Verma YL, Singh RK, Oh I, Chandra S (2014). Ionic liquid template assisted synthesis of porous nano-silica nails. RSC Adv..

[5] Vos ND, Maton C, Stevens CV (2014). Electrochemical stability of ionic liquids: General influences and degradation mechanisms. ChemElectroChem.

[6] Fedorov MV, Kornyshev AA (2014). Ionic liquids at electrified interfaces. Chem. Rev..

[7] Dou Q, Liu L, Yang B, Lang J, Yan X (2017). Silica-grafted ionic liquids for revealing the respective charging behaviors of cations and anions in supercapacitors. Nat. Commun..

[8] Shah FU, Gnezdilov OI, Gusain R, Filippov A (2017). Transport and association of ions in lithium battery electrolytes based on glycol ether mixed with halogen-free orthoborate ionic liquid. Sci. Rep..

[9] Cao S (2017). Green Brönsted acid ionic liquids as novel corrosion inhibitors for carbon steel in acidic medium. Sci. Rep..

[10] Silvester DS (2011). Recent advances in the use of ionic liquids for electrochemical sensing. Analyst.

[11] Shiddiky MJA, Torriero AAJ (2011). Application of ionic liquids in electrochemical sensing systems. Biosens. Bioelectron..

[12] Kwak K, Senthilkumar S, Pyo K, Lee D (2014). Ionic liquid of a gold nanocluster: A versatile matrix for electrochemical biosensors. ACS Nano.

[13] Pauliukaite R, Doherty AP, Murnaghan KD, Brett CMA (2008). Application of some room temperature ionic liquids in the development of biosensors at carbon film electrodes. Electroanalysis.

[14] Huddleston JG (2001). Characterization and comparison of hydrophilic and hydrophobic room temperature ionic liquids incorporating the imidazolium cation. Green Chem..

[15] Ao Y (2017). Fast selective homogeneous extraction of UO_2_^2+^ with carboxyl functionalised task-specific ionic liquids. Sci. Rep..

[16] Biswas T, Mahalingam V (2019). Efficient CO_2_ fixation under ambient pressure using poly(ionic liquid)-based heterogeneous catalysts. Sustainable Energy Fuels.

[17] Rantwijk FV, Sheldon RA (2007). Biocatalysis in Ionic Liquids. Chem. Rev..

[18] Wei D, Ivaska A (2008). Applications of ionic liquids in electrochemical sensors. Anal. Chim. Acta..

[19] Fujita K, Macfarlane R, Forsyth M (2005). Protein solubilising and stabilising ionic liquids. Chem. Commun..

[20] Zhang C, Zhang Z, Yang Q, Chen W (2018). Graphene-based electrochemical glucose sensors: Fabrication and sensing properties. Electroanalysis.

[21] Thenmozhi K, Narayanan SS (2017). Horseradish peroxidase and toluidine blue covalently immobilized leak-free sol-gel composite biosensor for hydrogen peroxide. Mater. Sci. Eng. C.

[22] Wan D, Yuan S, Li GL, Neoh KG, Kang ET (2010). Glucose biosensor from covalent immobilization of chitosan-coupled carbon nanotubes on polyaniline-modified gold electrode. ACS Appl. Mater. Interfaces..

[23] Manoj D, Theyagarajan K, Saravanakumar D, Senthilkumar S, Thenmozhi K (2018). Aldehyde functionalized ionic liquid on electrochemically reduced graphene oxide as a versatile platform for covalent immobilization of biomolecules and biosensing. Biosens. Bioelectron..

[24] Shen Y, Shen G, Zhang Y (2018). Voltammetric immunoassay for α-fetoprotein by using a gold nanoparticle/dendrimer conjugate and a ferrocene derived ionic liquid. Microchim. Acta..

[25] Bai Y, Xu T, Luong JHT, Cui H (2014). Direct electron transfer of glucose oxidase-boron doped diamond interface: A new solution for a classical problem. Anal. Chem..

[26] Olloqui-sariego JL (2015). Interprotein coupling enhances the electrocatalytic efficiency of tobacco peroxidase immobilized at a graphite electrode. Anal. Chem..

[27] Kavosi B, Salimi A, Hallaj R, Amani K (2014). A highly sensitive prostate-specific antigen immunosensor based on gold nanoparticles /PAMAM dendrimer loaded on MWCNTS/ chitosan/ionic liquid nanocomposite. Biosens. Bioelectron..

[28] Ghica ME, Pauliukaite R, Fatibello-filho O, Brett CMA (2009). Application of functionalised carbon nanotubes immobilised into chitosan films in amperometric enzyme biosensors. Sens. Actuators B Chem..

[29] Li P (2013). Direct electrochemistry of hemoglobin immobilized on the water-soluble phosphonate functionalized multi-walled carbon nanotubes and its application to nitric oxide biosensing. Talanta.

[30] Eguílaz M, Villalonga R, Rivas G (2018). Electrochemical biointerfaces based on carbon nanotubes-mesoporous silica hybrid material: Bioelectrocatalysis of hemoglobin and biosensing applications. Biosens. Bioelectron..

[31] Velusamy V (2017). Graphene dispersed cellulose microfibers composite for efficient immobilization of hemoglobin and selective biosensor for detection of hydrogen peroxide. Sens. Actuators B Chem..

[32] Cano RD (2018). Hemoglobin becomes electroactive upon interaction with surface-protected Au nanoparticles. Talanta.

[33] Xu H, Dai H, Chen G (2010). Direct electrochemistry and electrocatalysis of hemoglobin protein entrapped in graphene and chitosan composite film. Talanta.

[34] Ding L (2014). Phosphomolybdate@poly (diallyldimethylammonium chloride)-reduced graphene oxide modified electrode for highly efficient electrocatalytic reduction of bromate. J. Electroanal. Chem..

[35] Lee Y, Lee HJ, Jang A (2017). Amperometric bromate-sensitive sensor via layer-by-layer assembling of metalloporphyrin and polyelectrolytes on carbon nanotubes modified surfaces. Sens. Actuators B Chem..

[36] Rasheed PA, Pandey RP, Rasool K, Mahmoud KA (2018). Ultra-sensitive electrocatalytic detection of bromate in drinking water based on Nafion /Ti_3_C_2_T_x_ (MXene) modified glassy carbon electrode. Sens. Actuators B. Chem..

[37] Bates ED, Mayton RD, Ntai I, Davis JH (2002). CO_2_ capture by a task-specific ionic liquid. J. Am. Chem. Soc..

[38] Zhang M (2012). Schiff base structured acid – base cooperative dual sites in an ionic solid catalyst lead to efficient heterogeneous Knoevenagel condensations. Chem. Eur. J..

[39] Yue C, Su D, Zhang X, Wu W, Xiao L (2014). Amino-functional imidazolium ionic liquids for CO_2_ activation and conversion to form cyclic carbonate. Catal. Letters..

[40] Kiefer J, Fries J, Leipertz A (2007). Experimental vibrational study of imidazolium-based ionic liquids: Raman and Infrared spectra of 1-Ethyl-3- methylimidazolium Bis(trifluoromethylsulfonyl)imide and 1-Ethyl-3-methylimidazolium ethylsulfate. Appl. Spectrosc..

[41] Li Y (2010). Direct electrochemistry and electrocatalytic properties of hemoglobin immobilized on a carbon ionic liquid electrode modified with mesoporous molecular sieve MCM-41. Colloids Surf., B.

[42] Li J, Tang J, Zhou L, Han X, Liu H (2012). Direct electrochemistry and electrocatalysis of hemoglobin immobilized on polyacrylamide-P123 film modified glassy carbon electrode. Bioelectrochemistry.

[43] Wen Y, Wen W, Zhang X, Wang S (2016). Highly sensitive amperometric biosensor based on electrochemically-reduced graphene oxide-chitosan/hemoglobin nanocomposite for nitromethane determination. Biosens. Bioelectron..

[44] Liu H (2015). A novel nitrite biosensor based on the direct electrochemistry of hemoglobin immobilized on MXene-Ti_3_C_2_. Sens. Actuators B Chem..

[45] Zhong Y (2016). Electrochemically induced pitting corrosion of Ti anode: Application to the indirect reduction of bromate. Chem. Eng. J..

[46] Zhang Y (2014). Layer-by-layer construction of caterpillar-like reduced graphene oxide – poly (aniline- co-o-aminophenol)– Pd nanofiber on glassy carbon electrode and its application as a bromate sensor. Electrochim. Acta..

[47] Papagianni GG, Stergiou DV, Armatas GS, Kanatzidis MG, Prodromidis MI (2012). Synthesis, characterization and performance of polyaniline – polyoxometalates (XM_12_, X = P, Si and M = Mo, W) composites as electrocatalysts of bromates, *Sens*. Actuators B Chem..

[48] Shi E (2016). Electrochemical fabrication of copper-containing metal – organic framework films as amperometric detectors for bromate determination. Dalt. Trans..

[49] Li Q (2013). J. Synthesis of silver/multi-walled carbon nanotubes composite and its application for electrocatalytic reduction of bromate. Chem. Eng. J..

[50] Hassan SS (2013). Phosphomolybdate-doped-poly(3,4-ethylenedioxythiophene) coated gold nano particles: Synthesis, characterization and electrocatalytic reduction of bromate. Anal. Chim. Acta..

[51] Vilian ATE (2016). Immobilization of hemoglobin on functionalized multi-walled carbon nanotubes-poly-L-histidine-zinc oxide nanocomposites toward the detection of bromate and H_2_O_2_, *Sens*. Actuators B Chem..

[52] Li Y, Chen S, Thangamuthu R, Ali MA, Al-Hemaid FMA (2014). Preparation, characterization, and bioelectrocatalytic properties of hemoglobin incorporated multiwalled carbon nanotubes-poly-L-lysine composite film modified electrodes towards bromate. Electroanalysis.

[53] Palanisamy S (2016). Direct electrochemistry of immobilized hemoglobin and sensing of bromate at a glassy carbon electrode modified with graphene and β-cyclodextrin. Microchim. Acta..

