# Supplementary material for: Rationally designed naphthyl substituted amine functionalized ionic liquid platform for covalent immobilization and direct electrochemistry of hemoglobin
Source: Sci Rep. 2019 Jul 18;9:10428. doi: 10.1038/s41598-019-46982-3 (PMC6639313; doi:10.1038/s41598-019-46982-3)
Supplement: Supplementary file 1 — Rationally designed naphthyl substituted amine functionalized ionic liquid platform for covalent immobilization and direct electrochemistry of hemoglobin [file 41598_2019_46982_MOESM1_ESM.pdf]

Supplementary Information

Rationally designed naphthyl substituted amine functionalized ionic liquid platform for covalent immobilization and direct electrochemistry of hemoglobin

K. Theyagarajan, Duraisamy Saravanakumar, Sellappan Senthilkumar* and Kathavarayan Thenmozhi*

Department of Chemistry, School of Advanced Sciences, Vellore Institute of Technology, Vellore-632014, India.

*Corresponding Author: Tel: +914162202404; Email: senthilanalytical@gmail.com, senthilkumar.s@vit.ac.in (Sellappan Senthilkumar), kt.thenmozhi@gmail.com, k.thenmozhi@vit.ac.in (Kathavarayan Thenmozhi).

Chemicals and materials

Imidazole (≥99.0%), sodium hydride (NaH) (60%, in mineral oil), 1-(chloromethyl)naphthalene (≥97.0%), 3-bromopropylamine hydrobromide (98.0%), bis(trifluoromethane)sulfonimide lithium salt (Li-TFSI) (≥95.0%), terephthaloyl chloride (TP) (≥99.0%), hemoglobin porcine (lyophilized powder) were purchased from Sigma-Aldrich, India. Potassium bromate ($KBrO_3$) (99.5%), sodium phosphate monobasic (99%) and sodium phosphate dibasic (99%) were procured from Sisco Research Laboratories, India. Na_2HPO_4 and NaH_2PO_4 were used to prepare phosphate buffer solutions, which was used as supporting electrolyte and the pH was adjusted using aqueous NaOH. All other reagents were of analytical grade and used as obtained. Milli-Q water (18.2 MΩ cm resistivity at 25 °C) obtained from Millipore was used for the preparation of aqueous solutions.

Measurements

Fourier transform infrared (FTIR) spectral measurements were made using IR Affinity-1 Shimadzu FT-IR spectrophotometer using ATR method in the range between 4000 and 400 cm^{-1}. Nuclear magnetic resonance (NMR) spectral data were obtained from Bruker spectrometer (operated at 400 MHz for ^{1}H, 100 MHz for ^{13}C and 376 MHz for ^{19}F) in deuterated solvents DMSO-d_6 and $CDCl_3$ and the chemical shifts are referenced to TMS (δ = 0.00). The spectra were interpreted using TopSpin 3.5 software. High resolution mass spectrum (HRMS) was obtained on JEOL GC Mate-II spectrometer using electrospray ionization technique (EI, 70 eV). Cyclic voltammetry (CV) and amperometry studies were performed using CHI 620E electrochemical workstation (CH Instruments, Austin, Texas) and the electrochemical impedance spectroscopy (EIS) were performed on Autolab PGSTAT 204 (Metrohm Autolab, Netherlands). Conventional three electrode setup was used to perform electrochemical measurements. The modified or unmodified glassy carbon electrode (GCE) with a geometric working area of 0.071 cm^2 was used as the working electrode. Ag/AgCl (3

M KCl) was utilized as reference electrode, against which all the potentials are reported and Pt coil served as auxiliary electrode. Before the experiment, all electrochemical solutions were deaerated by purging continuous stream of high purity N_2 for at least 15 min.

Optimization of NpNH$_2$-IL and Hb loading during fabrication of biosensor

The optimization of the experimental parameters such as the amount of NpNH$_2$-IL and Hb were carried out. The concentration of NpNH$_2$-IL was varied from 0.06 to 0.14 mg (in 5 µL of methanol) with respect to 0.05 mg of Hb (in 5 µL of phosphate buffer) and the developed sensor has shown maximum current response for 0.1 mg of NpNH$_2$-IL. Further, the amount of NpNH$_2$-IL was kept constant (0.1 mg in 5 µL of methanol) and Hb concentrations were varied from 0.01 to 0.09 mg (in 5 µL of phosphate buffer), during which the electrode with 0.05 mg of Hb has shown maximum current response (Supplementary Figure. S9). Accordingly, the optimized loading for the effective biosensor fabrication was chosen to be 0.1 mg of NpNH$_2$-IL (in 5 µL of methanol) with 0.05 mg of Hb proteins (in 5 µL of phosphate buffer).

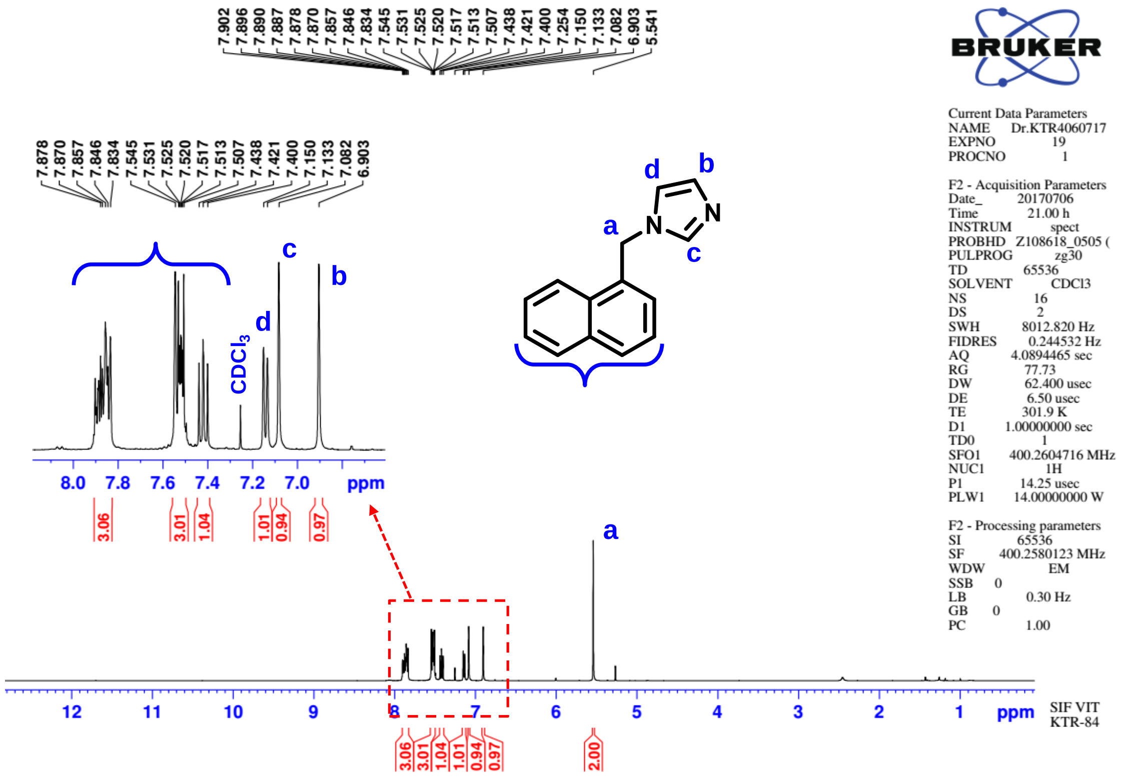

Supplementary Figure S1. ^{1}H NMR spectrum of 1-(naphthalen-1-ylmethyl)-1H-imidazole

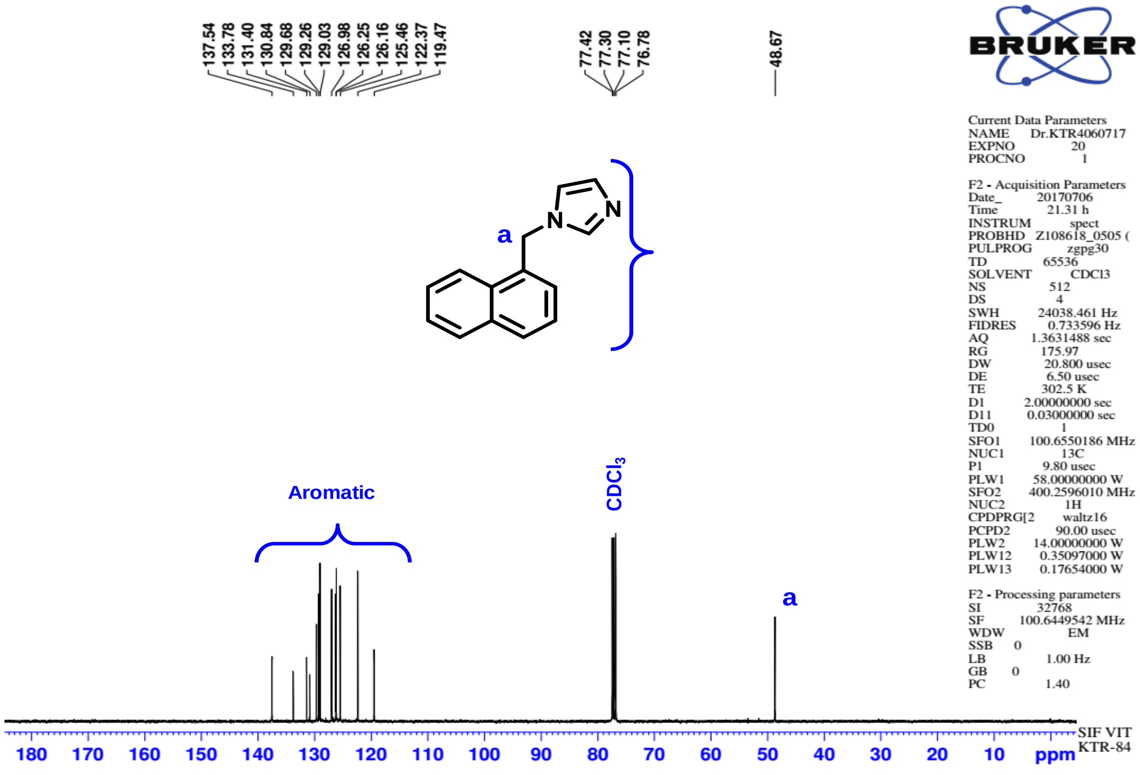

Supplementary Figure S2. ^{13}C NMR spectrum of 1-(naphthalen-1-ylmethyl)-1H-imidazole

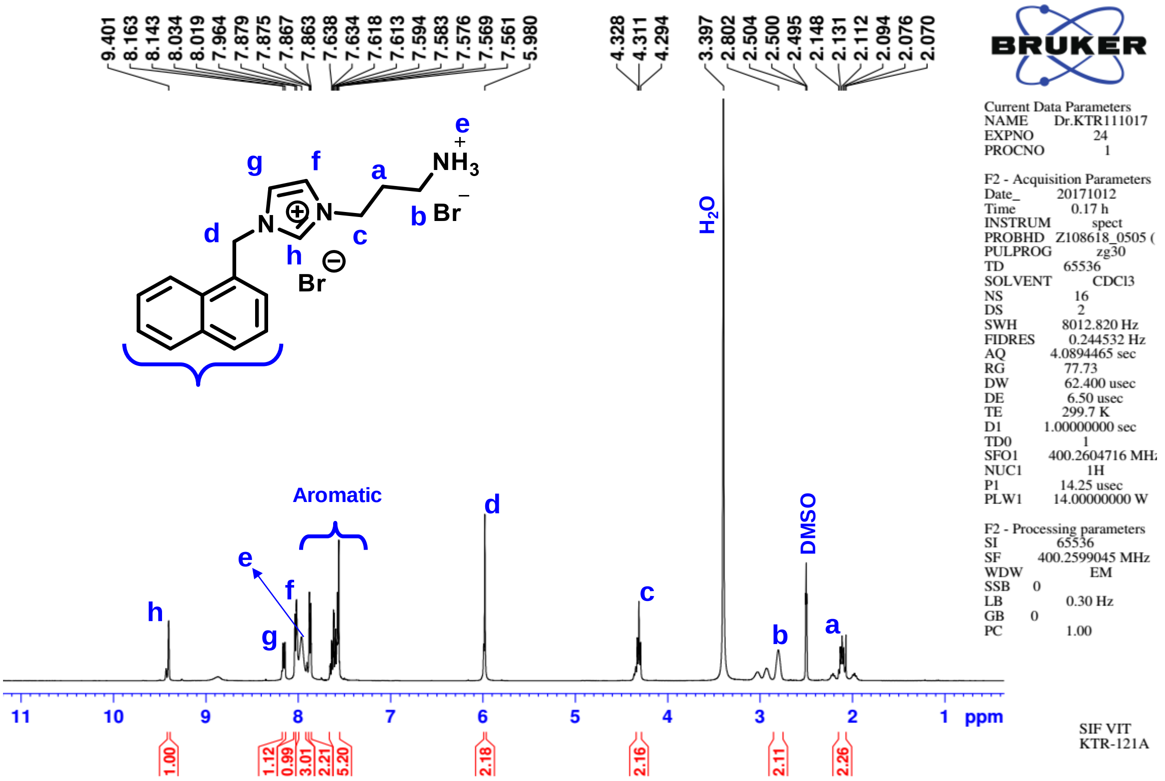

Supplementary Figure S3. ^{1}H NMR spectrum of 3-(3-ammoniopropyl)-1-(naphthalen-1-ylmethyl)-1H-imidazol-3-ium bromide (***2***)

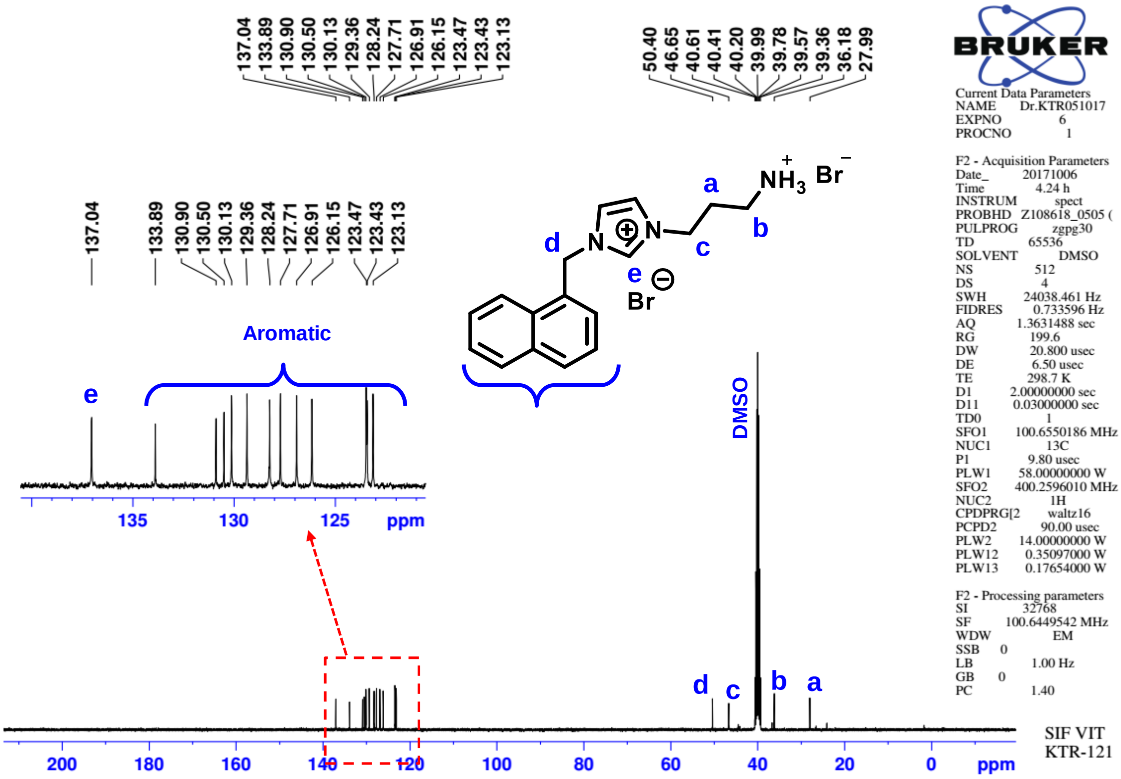

Supplementary Figure S4. ^{13}C NMR spectrum of 3-(3-ammoniopropyl)-1-(naphthalen-1-ylmethyl)-1H-imidazol-3-ium bromide (***2***)

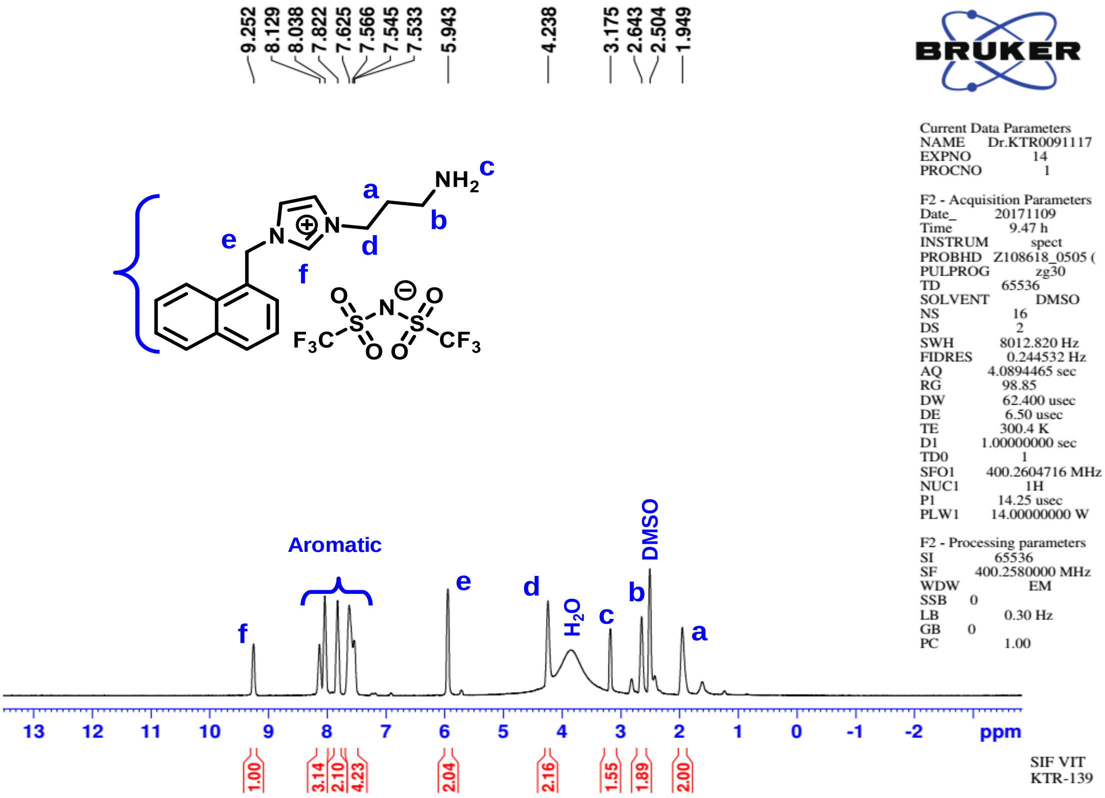

Supplementary Figure S5. ^{1}H NMR spectrum of 3-(3-aminopropyl)-1-(naphthalen-1-ylmethyl)-1H-imidazole-3-ium bis((trifluoromethyl)sulfonyl)amide **(NpNH2-IL) (*3*)**

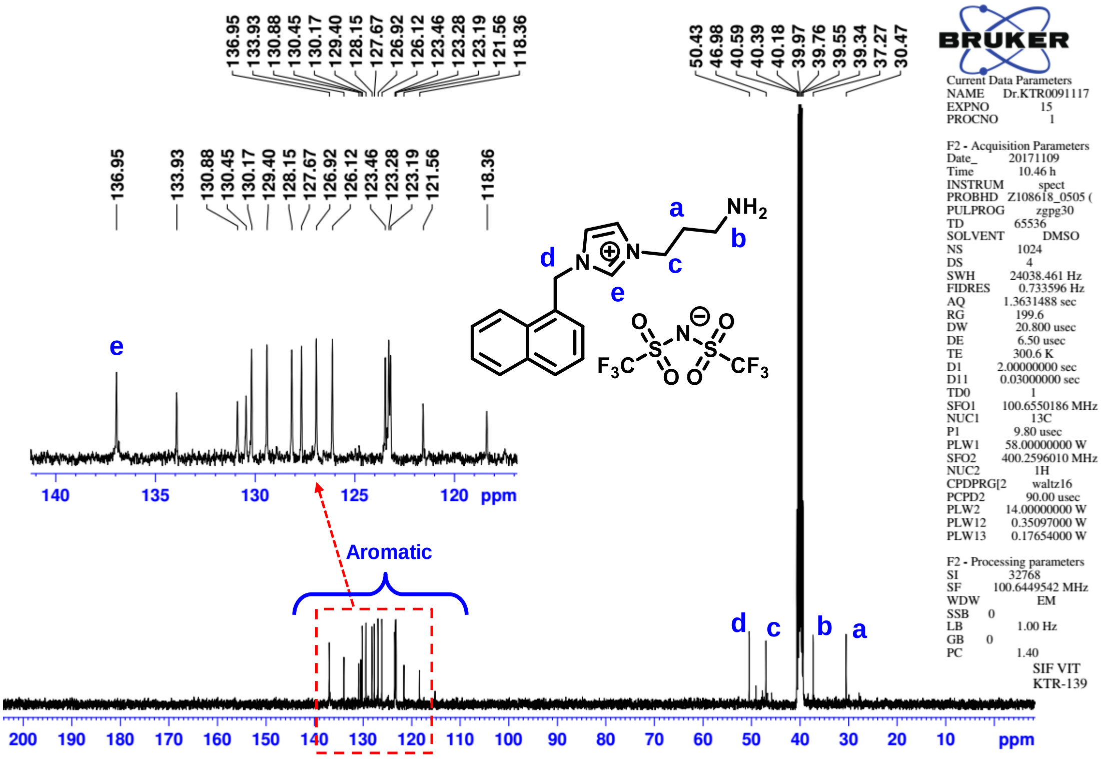

Supplementary Figure S6. ^{13}C NMR spectrum of 3-(3-aminopropyl)-1-(naphthalen-1-ylmethyl)-1H-imidazole-3-ium bis((trifluoromethyl)sulfonyl)amide **(NpNH2-IL) (*3*)**

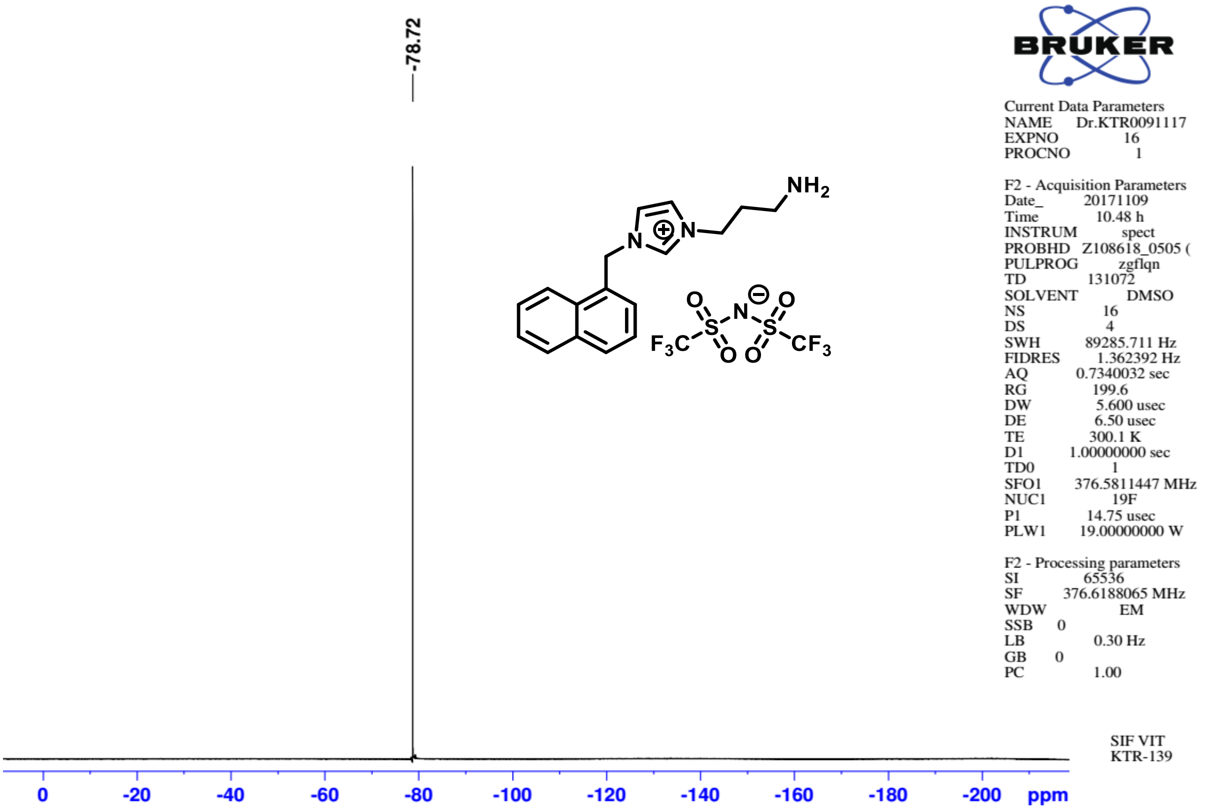

Supplementary Figure S7. ^{19}F NMR spectrum of 3-(3-aminopropyl)-1-(naphthalen-1-ylmethyl)-1H-imidazole-3-ium bis((trifluoromethyl)sulfonyl)amide **(NpNH$_2$-IL) (*3*)**

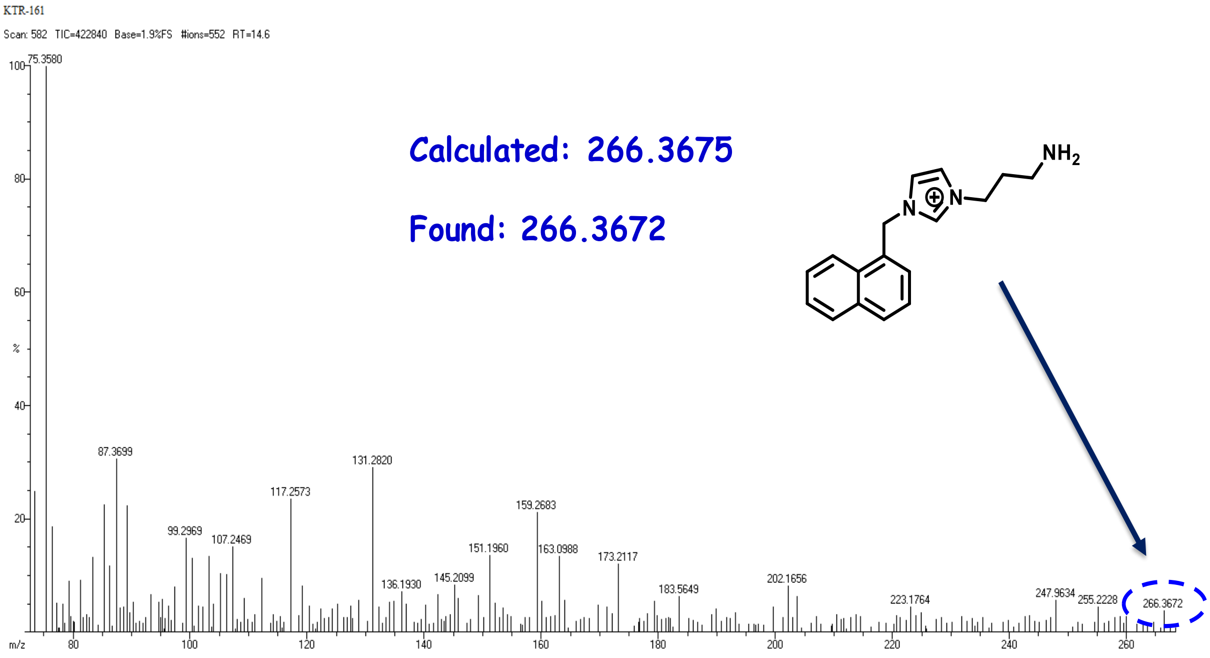

Supplementary Figure S8. HRMS spectrum of 3-(3-aminopropyl)-1-(naphthalen-1-ylmethyl)-1H-imidazole-3-ium bis((trifluoromethyl)sulfonyl)amide **(NpNH$_2$-IL) (*3*)**

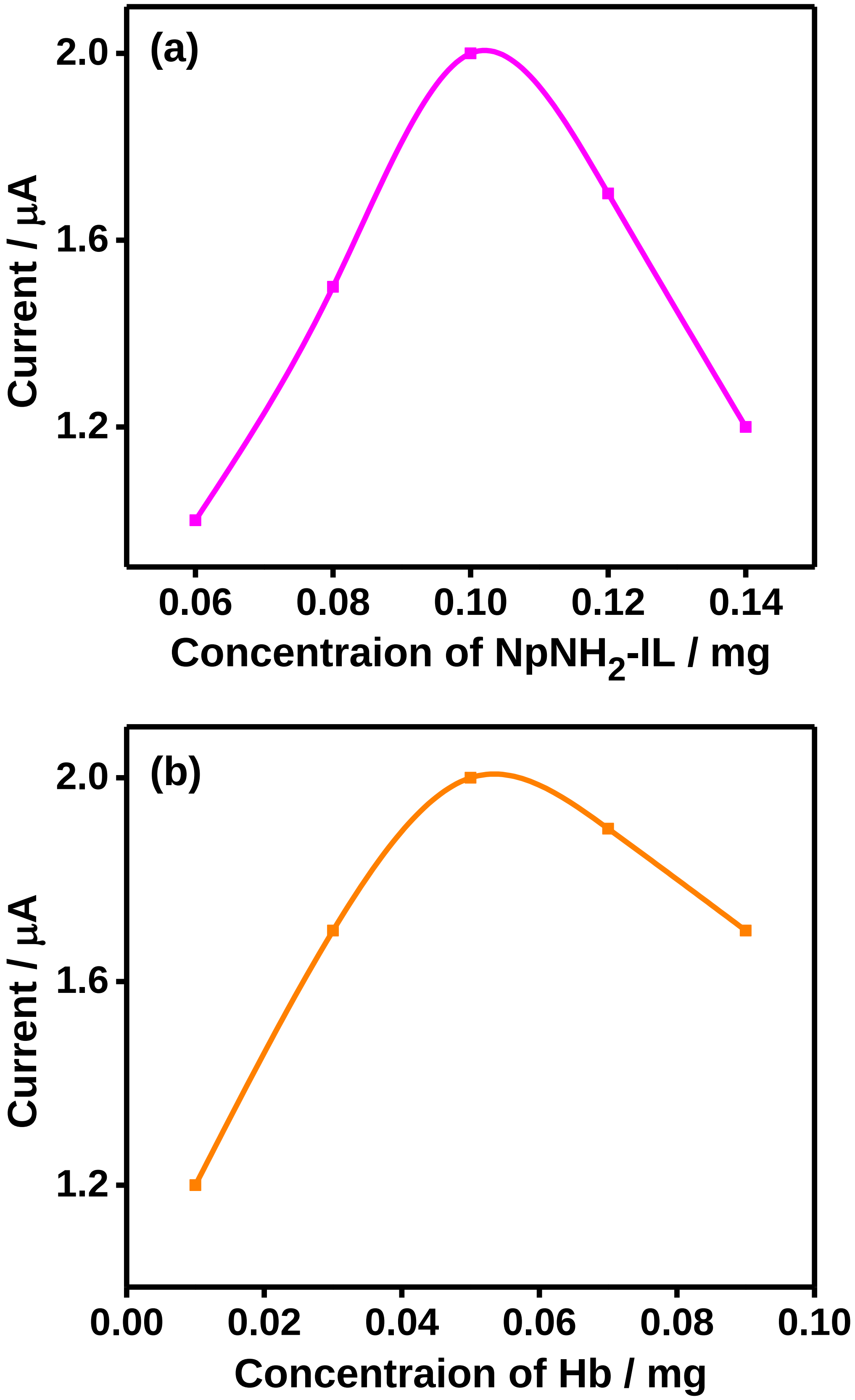

Supplementary Figure S9. Current response obtained during loading of different amounts of (a) $NpNH_2$-IL and (b) Hb in the fabrication of Hb-$NpNH_2$-IL/GCE.

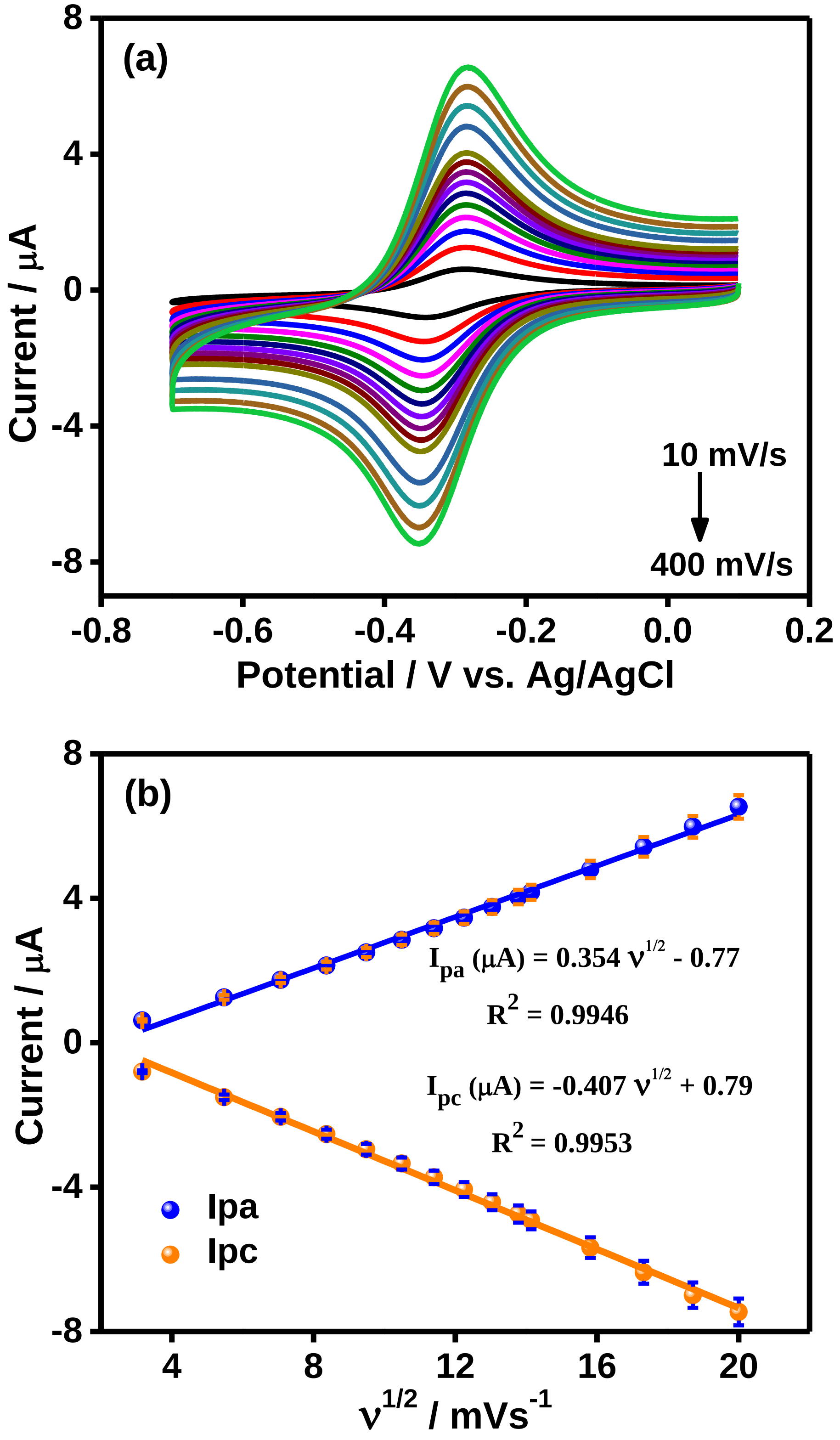

Supplementary Figure S10. (a) Cyclic voltammograms of Hb-NpNH_2-IL/GCE with increasing scan rate from 10 to 400 mV s^{-1}. (b) Plot of anodic and cathodic peak currents vs. square root of scan rate.

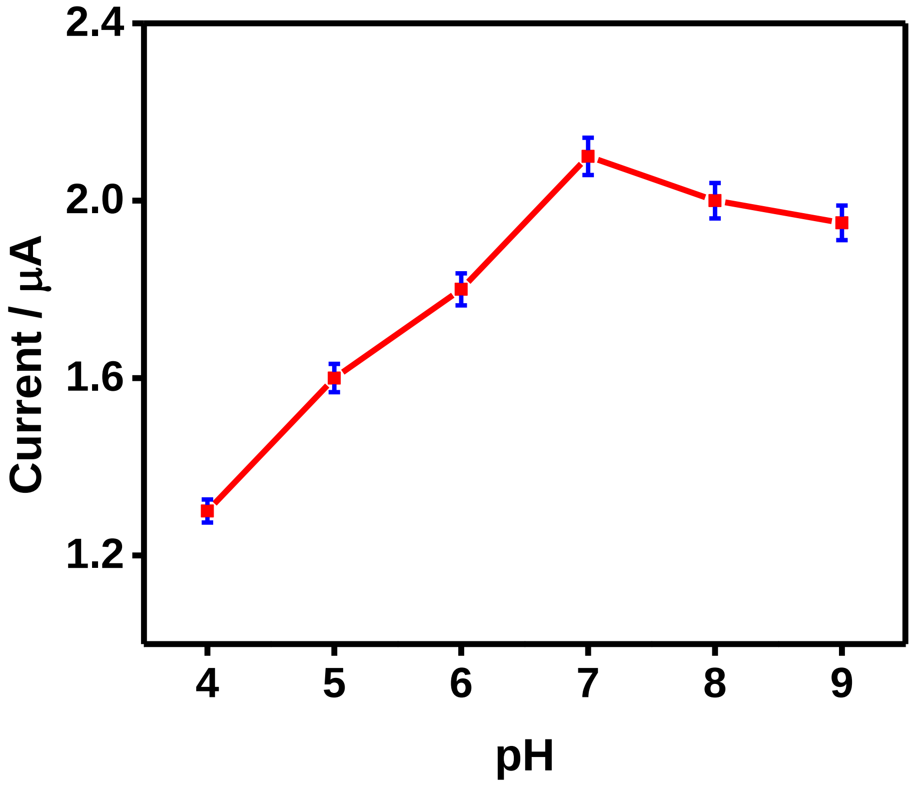

Supplementary Figure S11. Effect of pH on peak current of the Hb-NpNH$_2$-IL/GCE.

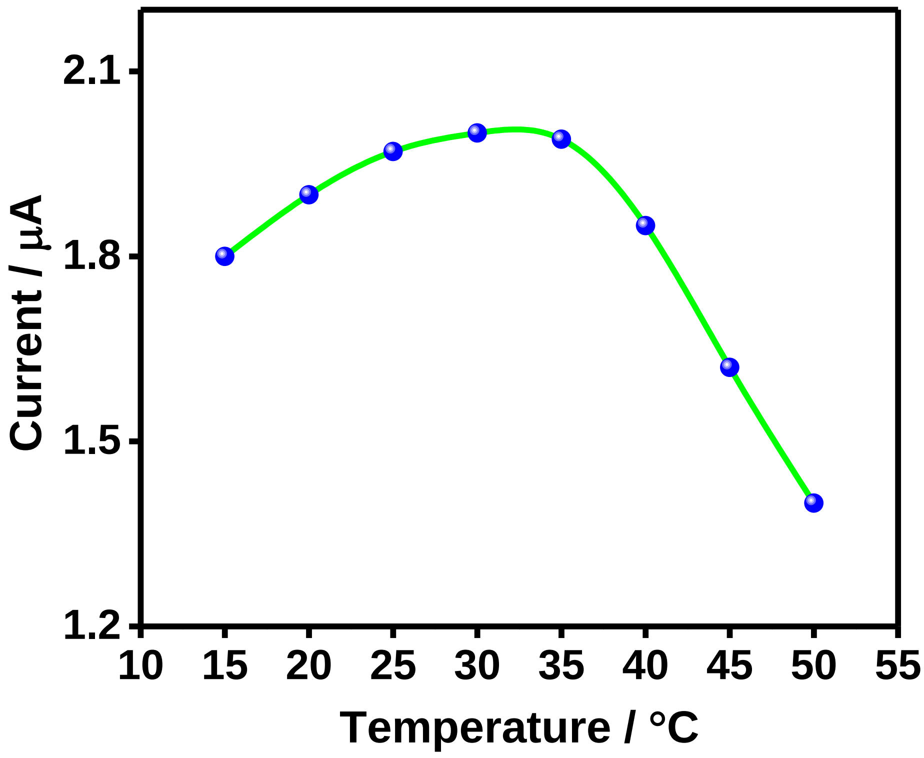

Supplementary Figure S12. Effect of temperature on the current response of Hb-NpNH$_2$-IL/GCE.

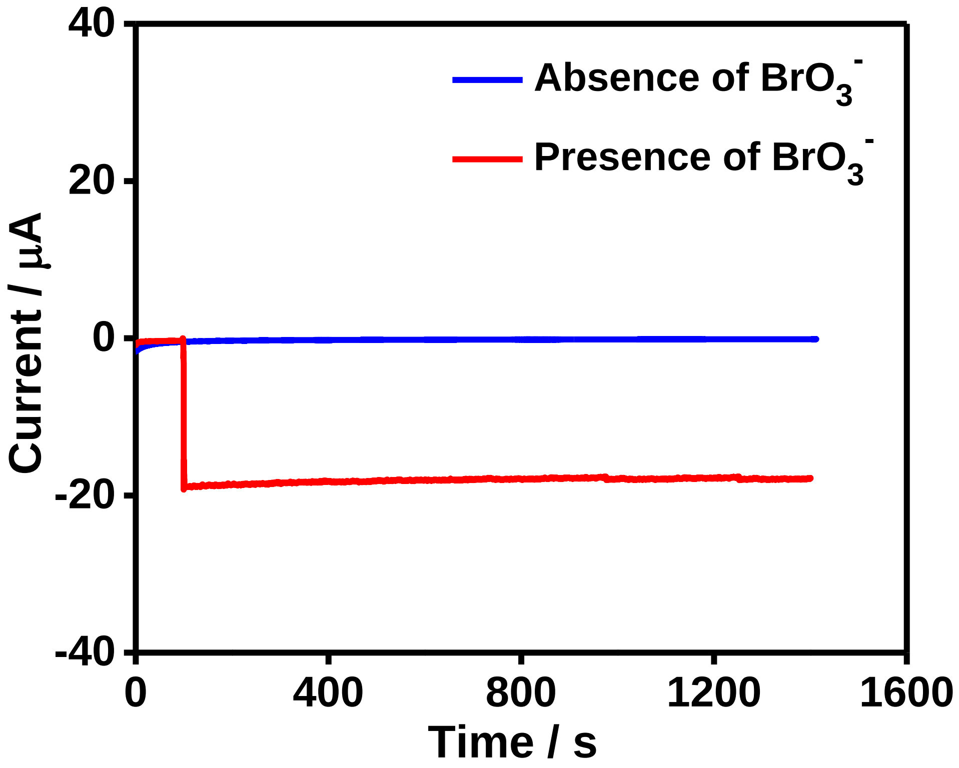

Supplementary Figure S13. Operational stability of Hb-NpNH$_2$-IL/GCE using amperometry at -0.4 V in the absence (blue) and presence (red) of 1.5 mM of bromate ions in a continuously stirred solution of N_2 saturated 0.1 M phosphate buffer solution (pH 7.0).

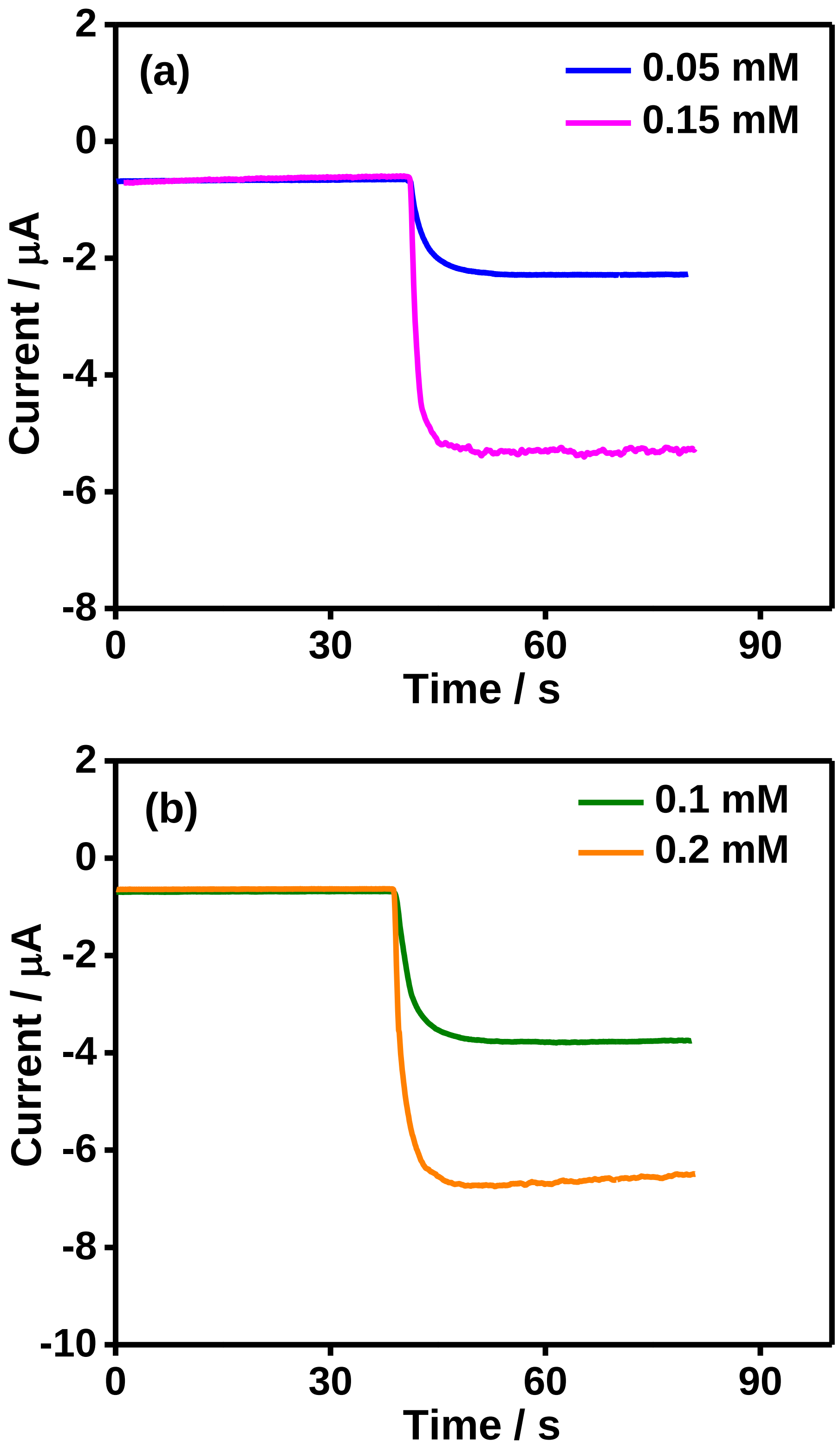

Supplementary Figure S14. Amperometric response of Hb-NpNH$_2$-IL/GCE (-0.4 V) towards the determination of bromate in (a) Tap water and (b) Drinking water. Electrolyte: N_2 saturated 0.1 M phosphate buffer solution (pH 7.0).
